# Considerations in characterizing real‐world data relevance and quality for regulatory purposes: A commentary

**DOI:** 10.1002/pds.4697

**Published:** 2018-12-05

**Authors:** Cynthia J. Girman, Mary E. Ritchey, Wei Zhou, Nancy A. Dreyer

**Affiliations:** ^1^ Patient‐reported Outcomes and Real‐world Evidence CERobs Consulting, LLC Chapel Hill North Carolina; ^2^ Epidemiology, Medical Devices, and Real‐World Evidence RTI—Health Solutions, Research Triangle Park Durham North Carolina; ^3^ Department of Pharmacoepidemiology, Center for Observational and Real‐world Evidence Merck & Co., Inc North Wales Pennsylvania; ^4^ Real‐World & Analytic Solutions IQVIA Cambridge Massachusetts

**KEY POINTS**

- Guidance on use of RWD for regulatory purposes should outline circumstances and level of acceptable validation/reliability for outcome, population, and exposure definitions given the research question and intended regulatory use.
- Representativeness of a population of interest for RWD should be considered in the context of specific research questions for regulatory purposes.
- Producing interpretable results necessitates valid, relevant research definitions for disease, outcomes, and treatments.
- A framework for assessing the utility and usefulness of RWD with respect to a specific research question and the intended regulatory purpose is needed for use by agencies, companies, and researchers around the world.

## 1 INTRODUCTION

The 21st Century Cures Act of 2016 provided a framework to the US Food and Drug Administration (FDA) to rapidly move treatments to patients.^1^ The increased acceptability of real‐world data (RWD) sources allows for innovative ways to study products and has the potential to reduce trial costs. Published papers provide guidance regarding data quality issues, reproducibility, and validity assessment.^2^ Rapid evolvement of electronic health records (EHRs) encourages greater consideration of their use in research.^1^, ^2^, ^3^, ^4^, ^5^, ^6^ For years, the FDA has relied on epidemiological studies of postapproval product safety using RWD^5^, ^6^ (eg, administrative claims and EHR) and for device effectiveness studies^4^; however, regulatory use for evaluating drug effectiveness has been rare. As part of the Prescription Drug User Fee Act (PDUFA VI),^3^ use of RWD is being considered for potential contributions to evaluating effectiveness and safety of new indications for approved products and to satisfy postapproval study requirements. Recently, the Duke Margolis Center for Health Policy held workshops and issued two paper on this topic.^5^, ^6^ The first paper focused on defining RWD as data routinely collected pertinent to patient health status and/or delivery of care, and the use of RWD in regulatory and clinical contexts.^5^

The second white paper from the October 1, 2018, workshop focused on data relevancy and quality, including cleaning, transforming, and linking RWD to characterize RWD sources as “fit for regulatory purpose.”^6^ These papers offer a practical “commonsense” high‐level view of primary data and methods considerations for RWD use from a regulatory perspective, facilitating discussion around regulatory uses of RWD within the research community and industry. However, salient points are missing from the papers and the RWD discussions among FDA, researchers, and industry. Here, we provide a commentary on the data considerations discussed in the white papers and highlight pertinent considerations with respect to RWD in the context of whether data are relevant, representative, and robust.

### 1.1 Data relevance

The recent white paper defines data relevance dimensions including representativeness of the population of interest, critical data field availability, accurate linking at the patient level with multiple data sources, and adequate sample size and follow‐up time to demonstrate expected treatment effects.^6^ Guidance from FDA on how to ensure RWD are fit for purpose and adequate to support regulatory decisions would be helpful on each dimension.

Determining if RWD is fit for regulatory purpose is a “*contextual exercise*” where the specific research question, regulatory use, and data characteristics drive what meaningful conclusions can be drawn.^6^ Covariates may be critical for one research question but not another. Exposures and outcomes should be well defined when part of the research question but may not be critical for natural history studies. There is no “one‐size‐fits‐all” approach, and critical data components should be evaluated for each research question and regulatory use.^7^ A framework is needed to guide choice and evaluation of critical data elements for specific research questions for regulatory use.

Representativeness of the population of interest is gauged in many ways. Recent FDA guidance on Patient Focused Drug Development suggests a statistical sampling approach be used to obtain patient experience data representative of the target population.^8^ However, most US real‐world databases use administrative claims or EHR for patients seeking medical attention. These RWD sources should be considered broadly representative of the population eligible for using most, if not all, new products and services. “Representativeness” should be assessed broadly in the context of likely product users with some diversity in geography, health status, and health care system as appropriate for the specific research question and regulatory context. While data linkage is likely to limit the eligible sample, it may be needed to increase the informative nature of RWD, especially with increasing evaluations to support precision medicine.

Sample size should be derived based on anticipated treatment effects for studies of treatment effectiveness or safety, whether comparative or not, to ensure appropriate precision of estimates. For rare diseases, there should be flexibility given data sparseness worldwide, as indicated in the FDA guidance on rare disease.^8^

Additional guidance would be useful regarding how “accurate linking” should be assessed since linking 100% of patients with administrative claims and EHR is impractical. Would FDA accept limited linked data if it was supplemental to cruder variables in the full dataset? Would a subset of 60% be adequate? In the context of probabilistic linkage, what level of certainty would constitute adequate linkage? Salience of linkable individuals to the specific research question should be considered in this determination and pre‐specified sensitivity analyses should help assess robustness of results and conclusions.^9^, ^10^

### 1.2 Data quality

Data quality should be considered in terms of validity, conformance, plausibility, and consistency.^11^ The acceptability of various degrees of accuracy and completeness depends on the specific research question and regulatory purpose. The white paper refers to data verification procedures, minimizing missing data, and consistency with source, often impractical given the anonymized nature of accessible data. RWD have proven valuable for specific purposes despite known limitations, when due attention is given to the adequacy of data elements, study design, and analysis. RWD used to support regulatory decisions must be of sufficient quality to ensure that it can be transformed to adequate and well‐controlled real‐world evidence.

Evaluations of data quality should be focused on fit‐for‐purpose design and methods, applying sensitivity analyses to support robustness and interpretation.^9^, ^10^ It is highly desirable to use a set of validated codes or algorithms (computable phenotypes) for critical fields, depending on study purpose. Decades of validation work in administrative claims have evaluated such algorithms relative to manual chart review.^12^ Now that the chart and data for research may be the same (ie, EHR), we need to understand how and when such validation should be conducted.^12^ Even if all available processes and SOPs for cleaning, transforming, and linkage are followed, overall data adequacy in the context of study and regulatory purpose should be assessed, preferably by a researcher experienced with RWD sources for regulatory decision making.

Missing data should be considered in the context of the impact on validity and generalizability of results. Whereas follow‐up data can be critical for certain purposes such as use of RWD as a comparative arm or concurrent/historic control group, missingness may be less critical for other purposes (eg, missing health outcomes may be less likely to affect results of a product utilization study than an outcomes study). That said, US RWD sources often are systematically missing follow‐up data due to turnover in health insurance plans and the US health care system's transient nature. Thus, a key consideration for any real‐world evidence research question is how much systematic loss of follow‐up data or other missing data would influence study conclusions.

### 1.3 Research framework

A fit‐for‐purpose framework starts with a well‐defined research question and an assessment of relevance and quality of specific critical data elements within the RWD source (Table 1). This might include assessing whether the population, outcomes, and treatments, as part of the PICOT definition^13^ of a well‐defined research question, can be validly and reliably defined using structured data (eg, diagnosis and procedure codes, laboratory tests, and pharmacy data) contained in RWD. If the critical data elements for a specific research objective can be defined in the RWD source, researchers might consider sample size and follow‐up time given the expected effect size, whether validation is needed for critical data elements, and what level of missing data can be tolerated (Table 2), given the specific research question and regulatory use. With data linkage, these considerations would be applicable to the separate data sources and the linked data.

**Table 1:** Type of structured data in RWD sources possibly needed to define elements of research question, depending on research question

What is the research question?	
In specifying the research question, include the relevant data elements^13^ such as *population*, *intervention* and *comparator* (*treatments*), and *outcome*, as applicable (eg, to assess effects of intervention compared with comparator treatment on the incidence of outcome over 2 years (*timing*) in a population of patients with disease).	
For Research Involving This Data Element:	Type of Structured Data in RWD Sources Possibly Needed to Define Data Elements*
Population	Diagnosis codes Procedure codes Laboratory values Pharmacy data (rarely)
Intervention and comparator (treatments; drug, biologic, or medical device)	Pharmacy data Procedure codes
Outcome	Diagnosis codes Procedure codes Laboratory values

Abbreviation: RWD, real‐world data.

* Specific type of data for each research data element depends on the research question

**Table 2:** Considerations for choosing RWD sources for research studies

	Key Considerations
Adequate sample size	□ RWD addresses the scientific question with sufficient confidence. □ There are sufficient persons, follow‐up time, and relevant observations to address the scientific question. □ Absent specific feasibility numbers, the crude prevalence can be applied to the total person‐lives in the database to crudely estimate sample size (without applying entry criteria).
Research data element definitions and validation	□ Essential data elements are coded consistently in the RWD health care system (codes capture the research data fields, eg, disease, outcome, treatment, critical covariates, if relevant, adequately). □ Systematic errors (eg, downcoding or upcoding) in the study population and essential data element definitions are identified and minimized and pre‐specified sensitivity analyses can assess potential impact, if possible. □ Definitions for essential data elements (eg, population and outcome) are unlikely to result from “screening” or “rule out” of a specific diagnosis in clinical practice. □ Needed coding algorithms (eg, computable phenotypes) are available and validated for essential data elements. □ If additional validation is needed, given the research purpose and regulatory decision, then it can be performed within the data source. □ Covariates or confounders are available that are critical to the research question. □ (*If needed*) *variables that correlate highly with key missing confounders are available and can be used instead*.
Missingness and completeness	□ Consideration has been made regarding essential elements of the research question that may be systematically missing due to patients seeking care out of network or changes in insurance coverage and whether the outcome can be captured reliably over time within the RWD source. □ Level of systematic error will not substantially affect study interpretation. □ Discrepancies between different sources of linked data (claims and EHR) for the data elements needed for specific research question will not affect interpretation of the study results. □ In combining data from multiple health care systems, different coverage policies or benefit designs do not affect ability to address the research question.

Abbreviation: RWD, real‐world data.

Preliminary data extraction may be performed to crudely determine number of patients and median follow‐up time in the specific RWD source. Very small effect sizes may be difficult to address with precision in RWD sources due to potential for bias. Research with larger expected effect sizes can often be addressed with RWD, with careful attention to appropriate design and methods. At a very high level, one can apply the crude estimate of disease or exposure prevalence (whichever is smaller) to the number of lives covered in a database to better understand adequate sample size.

A framework to assess usefulness of RWD in the context of specific research questions and intended regulatory purpose, along with published reporting guidelines,^9^, ^14^, ^15^ could significantly help identify major components of well‐designed studies in RWD to support specific product effectiveness and safety research questions for regulatory purposes.

## 2 CONCLUSIONS

Recent papers on use of RWD for regulatory purposes have initiated discussions among regulators, industry, and researchers on practical considerations of RWD relevance and quality. Beyond availability of data fields, valid definitions of components of research questions are crucial. More guidance is needed on what constitutes acceptable evidence of validation for critical data elements given the clinical research question and intended regulatory use. Besides FDA, other agencies are also exploring the appropriate usage of RWD in regulatory decisions. Understanding how to use RWD and whether they are “fit for purpose” is helpful for regulatory agencies, industry, and researchers around the world.
